# Supplementary material for: Ferroptosis-Inhibitory Difference between Chebulagic Acid and Chebulinic Acid Indicates Beneficial Role of HHDP
Source: Molecules. 2021 Jul 15;26(14):4300. doi: 10.3390/molecules26144300 (PMC8303713; doi:10.3390/molecules26144300)
Supplement: Supplementary file 1 [file molecules-26-04300-s001.zip › Supplementary Materials/Supplementary Materials 1. Dose responsecurves.pdf]

Supplementary 1. Dose response curves Figures S1–S3

Ferroptosis-Inhibitory Difference between Chebulagic Acid and Chebulinic Acid Indicates Beneficial Role of HHDP

Lin Yang[1], Yangping Liu[2], Wenhui Zhang[3], Yujie Hua[3], Ban Chen[3], Quanzhou Wu[3], Dongfeng Chen[1], Shuqin Liu[3], Xican Li[*,3]

1 School of Basic Medical Science, Guangzhou University of Chinese Medicine, Waihuan East Road No. 232, Guangzhou Higher Education Mega Center, Guangzhou, China, 510006.

2 The Fourth Clinical Medical College, Guangzhou University of Chinese Medicine, Waihuan East Road No. 232, Guangzhou Higher Education Mega Center, Guangzhou, China, 510006.

3 School of Chinese Herbal Medicine, Guangzhou University of Chinese Medicine, Guangzhou, China, 510006.

* Correspondence: lixc@gzucm.edu.cn (X.L.); Tel: +86-20-39358076; Fax: +86-20-38892690

Note: This Supporting information provides the original data of **Table 1** in the main text. All data marked with an asterisk (*) are mentioned in **Table 1** in the main text.

1. PTIO•-inhibition assay

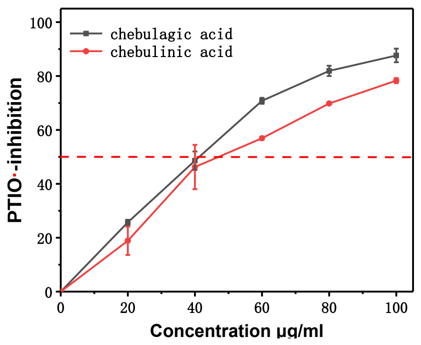

Figure S1a: The dose response curves of chebulagic acid and chebulinic acid in PTIO•-inhibition assay (pH7.4). Each value is expressed as mean ± SD (n = 3).

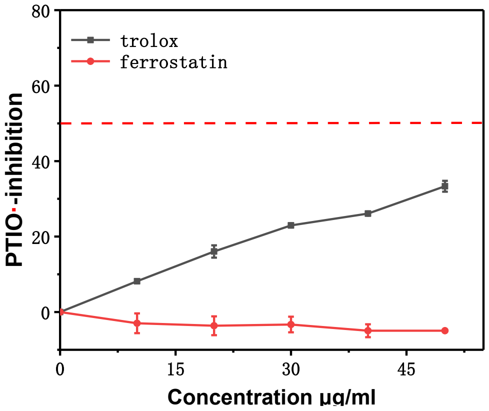

Figure S1b: The dose response curves of Trolox, and Ferrostatin-1 in PTIO•-inhibition assay (pH7.4). Each value is expressed as mean ± SD (n = 3).

Table S1. The comparison of IC_{50} values of chebulagic acid, chebulinic acid, and positive controls in PTIO•-inhibition assay (pH7.4).

	Mean±SD μg/mL	Mean±SD μM
chebulagic acid	43.5±1.6	40.1±1.5*
chebulinic acid	54.0±4.7	56.4±5.0*
Trolox	77.7±3.7	310.3±14.8*
Ferrostatin-1	n.d.	n.d.*

IC_{50} value was defined as the concentration of 50% superoxide anion radical inhibition, and calculated by linear regression which was analyzed by Origin 6.0 professional software. All data marked with an asterisk (*) are mentioned in **Table 1** in the main text.

2. Fe^{3+}-reducing antioxidant power (FRAP) assay

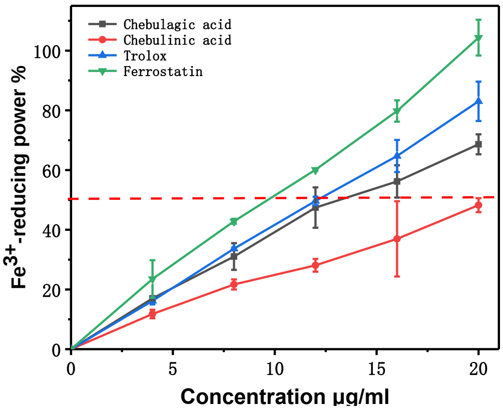

Figure S2: The dose response curves of chebulagic acid, chebulinic acid, Trolox, and Ferrostatin-1 in FRAP assays. Each value is expressed as mean ± SD (n = 3).

Table S2. The comparison of IC_{50} values of chebulagic acid, chebulinic acid, and positive controls in FRAP assay.

	Mean±SD μg/mL	Mean±SD μM
chebulagic acid	13.9±1.2	14.4±0.1*
Tannic acid	21.4±2.2	12.8±1.1*
Trolox	12.2±0.6	48.7±2.5*
Ferrostatin-1	9.5±0.4	36.3±1.5*

IC_{50} value was defined as the concentration of 50% superoxide anion radical inhibition, and calculated by linear regression which was analyzed by Origin 6.0 professional software. All data marked with an asterisk (*) are mentioned in **Table 1** in the main text.

3. DPPH•-inhibition assay

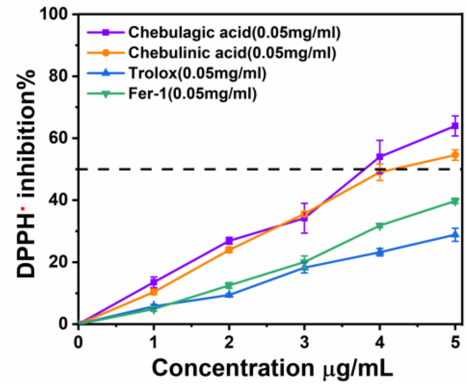

Figure S3: The dose response curves of chebulagic acid, chebulinic acid, Trolox, and Fer-1 in DPPH•-inhibition assay. Each value is expressed as mean ± SD (n = 3).

Table S3. The comparison of IC_{50} values of chebulagic acid, chebulinic acid and positive control in DPPH•-inhibition assay.

	Mean±SD μg/mL	Mean±SD μM
Chebulagic acid	3.8± 0.1	4.0± 0.1*
Chebulinic acid	4.3± 0.4	4.5± 0.4*
Trolox	8.7± 1.2	34.9± 4.9*
Fer-1	6.2± 0.2	23.5± 0.7*

IC_{50} value was defined as the concentration of 50% superoxide anion radical inhibition and calculated by linear regression which was analyzed by Origin 6.0 professional software. Fer-1, Ferrostatin-1. All data marked with an asterisk (*) are mentioned in **Table 1** in the main text.

4. ABTS•-inhibition assay

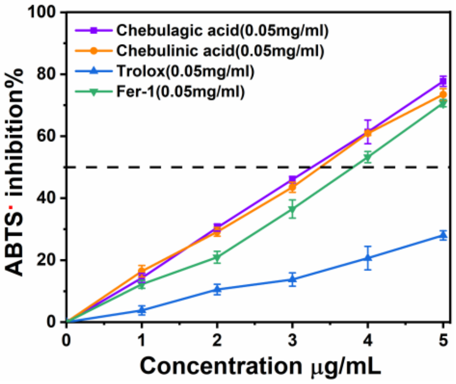

Figure S4: The dose response curves of chebulagic acid, chebulinic acid, Trolox, and Fer-1 in ABTS•-inhibition assay. Each value is expressed as mean ± SD (n = 3).

Table S4. The comparison of IC_{50} values of chebulagic acid, chebulinic acid and positive control in ABTS•-inhibition assay.

	Mean±SD μg/mL	Mean±SD μM
Chebulagic acid	3.3± 0.0	3.4± 0.0*
Chebulinic acid	3.4± 0.1	3.5± 0.1*
Trolox	8.9± 0.2	35.7± 0.8*
Fer-1	3.9± 0.2	14.9± 0.8*

IC_{50} value was defined as the concentration of 50% superoxide anion radical inhibition and calculated by linear regression which was analyzed by Origin 6.0 professional software. Fer-1, Ferrostatin-1. All data marked with an asterisk (*) are mentioned in **Table 1** in the main text.
