# Supplementary figures and images for: Ferroptosis-Inhibitory Difference between Chebulagic Acid and Chebulinic Acid Indicates Beneficial Role of HHDP
Source: Molecules. 2021 Jul 15;26(14):4300. doi: 10.3390/molecules26144300 (PMC8303713; doi:10.3390/molecules26144300)

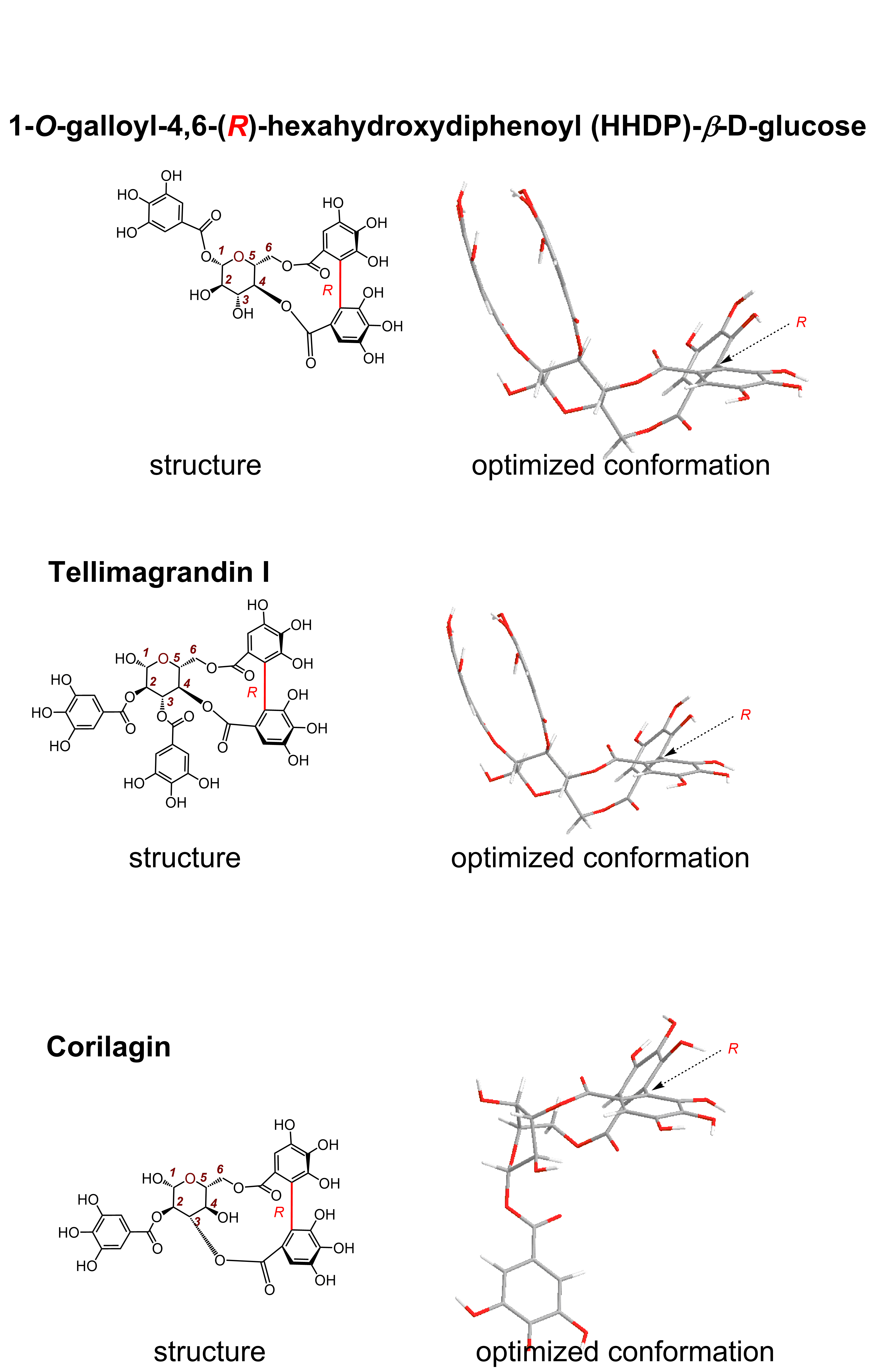

Supplement: Supplementary file 1 [file molecules-26-04300-s001.zip › Supplementary Materials/Supplementary Materials 2. Extra structures.png]
